# A new spray‐based method for the in‐vitro development of dry‐surface biofilms

**DOI:** 10.1002/mbo3.1330

**Published:** 2023-01-11

**Authors:** Esther Christine, Claude Olive, Myriam Louisin, Moustapha Dramé, Karine Marion‐Sanchez

**Affiliations:** ^1^ Department of Bacteriology Hygiene and Environment Laboratory, CHU Martinique CS 90632 Fort‐de‐France Cedex Martinique; ^2^ Department of Clinical Research and Innovation CHU Martinique CS 90632 Fort‐de‐France Cedex Martinique; ^3^ Department of Hospital Hygiene CHU Martinique CS 90632 Fort‐de‐France Cedex Martinique; ^4^ Pathogenesis and Control of Chronic and Emerging Infections, Université de Montpellier, Université des Antilles, Inserm, Etablissement Français du Sang CHU Martinique Montpellier France

**Keywords:** artificial saliva, dry‐surface biofilm, *Enterobacter cloacae*, *Klebsiella pneumoniae*, *Staphylococcus aureus*

## Abstract

The inanimate environment immediately surrounding the patient in healthcare facilities is a reservoir of microorganisms embedded in dry‐surface biofilms (DSB). These biofilms, first highlighted in 2012, are increasingly studied, but currently available in‐vitro models only allow for the growth of semi‐hydrated biofilms. We developed a new in‐vitro method under actual dehydration conditions based on the hypothesis that surface contamination is mainly due to splashes of respiratory secretions. The main objective of this study was to show that the operating conditions we have defined allowed the growth of DSB with a methicillin resistant *Staphylococcus aureus* strain. The second objective was to show that extended‐spectrum beta‐lactamase‐producing Enterobacteriaceae, that is, *Klebsiella pneumoniae* and *Enterobacter cloacae* were also able to grow such biofilms under these conditions. Monobacterial suspensions in sterile artificial saliva (SAS) were sprayed onto polyethylene surfaces. Nutrients and hydration were provided daily by spraying SAS enriched with 20% of Brain Heart Infusion broth. The primary outcome was mean surface coverage measured by image analysis after crystal violet staining. The method applied to *S. aureus* for 12 days resulted in reproducible and repeatable DSB consisting of isolated and confluent microcolonies embedded in extracellular polymeric substances as shown in scanning electron microscopy images. Similar DSB were obtained with both Enterobacteriaceae applying the same method. No interspecies variation was shown between the three strains in terms of surface coverage. These first trials are the starting point for a 3‐year study currently in process.

## Graphical abstract

Bacteria can persist in the hospital environment inside dry biofilms which grow on inanimate surfaces surrounding the patients. We developed a new method to produce dry‐surface biofilms using the spraying of enriched artificial saliva which mimics the dispersion of respiratory secretions. The method created dehydrated biofilms of methicillin resistant *Staphylococcus aureus* and Enterobacteria under real‐world conditions.

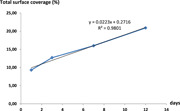

## 1 INTRODUCTION

The inanimate environment immediately surrounding a patient is a well‐recognized source of cross‐transmission of microorganisms, including multiresistant bacteria. Indeed, microorganisms disseminated by the patient can remain in his environment for a long time despite regular cleaning and be transmitted to another patient, either by direct contact or by the hands of healthcare workers (Chowdhury et al., 2018; Otter et al., 2013; Weber et al., 2013). In 2012, Vickery et al. highlighted the persistence of methicillin resistant *Staphylococcus aureus* (MRSA) inside biofilms on dry clinical surfaces in intensive care units (Vickery et al., 2012). Three years later, such biofilms have been referred to as “dry‐surface biofilm” (DSB) (Hu et al., 2015). Unlike fully hydrated biofilms which have been studied for about 40 years (Costerton et al., 1987), the level of hydration in DSB is reported to be 57%–72% but extracellular polymeric substances are thicker (Almatroudi et al., 2018). It has been hypothesized that DSB develops through regular contact with surfaces by the hands of healthcare workers or the patient himself. The hydration phases would result from the diffusion of biological fluids (sweat, blood, urine, sputum, and so forth) or the regular use of chemicals when cleaning surfaces (Hu et al., 2015).

To study such biofilms, five laboratory models have been presented in the literature to date. The first, based on the dynamic CDC Biofilm Reactor (Goeres et al., 2005), was described by Almatroudi (Almatroudi et al., 2015). It alternates daily hydration phases of several hours on polycarbonate coupons with drying phases of 42–66 h. It was then modified by two authors. Nkemngong first grew standard hydrated biofilms using the same reactor for 48 h on borosilicate glass coupons which were then dried for 24–120 h (Nkemngong et al., 2020). Amaeze et al. (2020) replaced the CDC continuous‐stirred tank reactor with 24‐well tissue culture plates and created both a dynamic and a static model. Ledwoch et al. (2019) also alternated hydration and desiccation phases, each for 48 h, but used a sedimentation protocol on stainless‐steel coupons inside cell culture plates. Watson et al.(2018), on the other hand, chose the drip flow reactor (Goeres et al., 2009) to first grow hydrated biofilms on stainless‐steel coupons in 54 h and then let them dry for 48 h using an aquatic air pump. All of these models include a significant growth phase under fully hydrated conditions so that they lead to the development of semidehydrated biofilms. In addition, they all use rich culture media (Tryptic Soy Broth) to provide nutrients and hydrate the biofilms, which distances them from real‐life conditions.

We report here the design and feasibility assessment of a spray‐based method for the growth of dehydrated biofilms under real‐life conditions, as close as possible to those encountered in a general intensive care unit. The aim of the study was twofold: (1) to show that the operating conditions we have defined allowed the growth of reproducible and repeatable *Staphylococcus aureus* DSBs which looked like environmental DSBs; (2) to apply the method to two extended‐spectrum beta‐lactamase‐producing Enterobacteriaceae (ESBL‐PE) and show that they were also capable of forming DSBs similar to those obtained with the MRSA strain.

## 2 MATERIAL AND METHOD

Experiments were performed at room temperature, that is, 22°C ± 2°C with 55%–65% relative humidity. Such conditions are maintained inside the entire building where both laboratories and intensive care units are located, by an air handling unit to control the excessive temperature and humidity of the tropical climate. Moreover, experiments were performed under a laminar flow hood (MSC 1.2 PSM II; Thermo Fischer Scientific) to avoid external contaminations during the growth of the monobacterial biofilms.

All sets of experiments included an untreated control, that is, a sterile 55 mm PE‐LD Petri dish left open under the laminar flow during hydration and gradual desiccation phases and closed during the dry‐holding phase.

### 2.1 Bacterial strains

The method was first developed with a clinical strain of MRSA isolated from a blood sample of a patient with catheter‐related bacteremia. It was then applied to two ESBL‐PE, *Klebsiella pneumoniae* and *Enterobacter cloacae*, isolated from inanimate surfaces, respectively, a sink and a patient call button in the old disused intensive care unit of our hospital. These strains were part of our institutional collection and were stored at −70°C on glass beads. Each strain was tested separately.

### 2.2 Sterile artificial saliva

The solution chosen to dilute the inoculum, provide nutrients, and hydrate the biofilms was sterile artificial saliva (SAS) prepared according to the Artisial® drug formulation (Biocodex) (Table A1). Each strain was first tested for survival and growth in this medium (Table A2).

### 2.3 Inoculum

Each strain was first cultivated in Brain Heart Infusion (BHI) broth for 24 h at 37°C. The resulting suspension (containing approximately 10^9^ CFU/mL) was then diluted 1:1000 in SAS to obtain a final suspension of approximately 10^6^ CFU/mL, verified by serial dilutions and spreading on Plate Count Agar. On Day 0, the inoculum was sprayed once on the inner surface of sterile 55 mm PE‐LD Petri dishes.

### 2.4 Nutrition and hydration

Nutrition and hydration of attached cells were carried out once a day, from Day 1 to endpoint, with two successive sprays of SAS enriched with 20% of BHI broth.

### 2.5 Spraying technique

**Figure 1:**
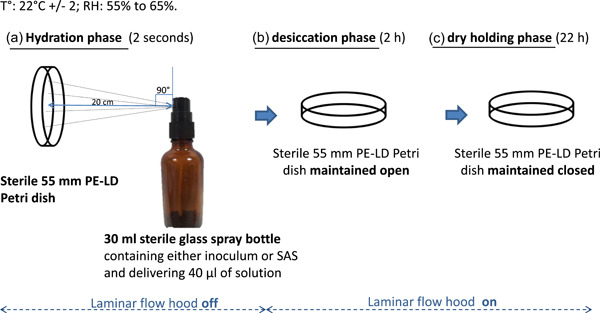
Experimental protocol. Day 0: one spray of inoculum on the inner surface of the Petri dish. From Day 1 to Day 12: nutrition and hydration of attached cells once a day, with two successive sprays of sterile artificial saliva (SAS).

The inoculum or enriched SAS was poured into a 30‐mL sterile glass spray bottle (Avalon Cosmetic Packaging). The laminar flow was stopped. Petri dishes were held vertically facing the spray, at approximately 20 cm of the spray nozzle which was placed perpendicularly to the center of the surface. They were manually sprayed and left open for 24 h under laminar flow. Each spray delivered 40 µL of enriched SAS. The experimental protocol is illustrated in Figure 1.

### 2.6 Endpoint setting

The endpoint of experiments was set to Day 12 in accordance with the literature (Almatroudi et al., 2015; Amaeze et al., 2020; Ledwoch et al., 2019) and was confirmed by a time‐course experiment with the MRSA strain in which duplicate biofilm samples were analyzed on Days 1, 3, 7, and 12.

### 2.7 Biofilm analysis

At the endpoint, Petri dishes were rinsed with sterile distilled water and the attached biomass was stained with Crystal Violet (RAL Diagnostics). One‐quarter of the surface was randomly selected online (https://www.random.org), observed in its entirety by light microscopy (×500, immersion objective), and 20 photographs were taken (ICC50E; Leica); 10 of these were randomly selected online for the calculation of the MSC by image analysis using Scion Images software (Scion Corporation) (Table A3).

**Figure 2:**
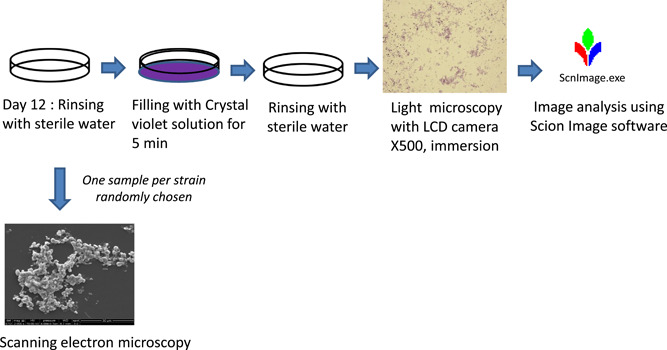
Biofilm analysis.

In addition, one sample per strain was randomly chosen and observed by scanning electron microscopy (SEM) (FEI Quanta 250 FEG; ThermoFisher Scientific). The analysis method is illustrated in Figure 2.

### 2.8 Data analysis

Quantitative data were expressed as mean ± standard deviation (SD). To examine for between‐run, within‐run, and interspecies variations, an analysis of variance (ANOVA) was performed (release 9.4; SAS Software).

## 3 RESULTS AND DISCUSSION

### 3.1 The main objective: To grow DSBs with the MRSA strain

**Figure 3:**
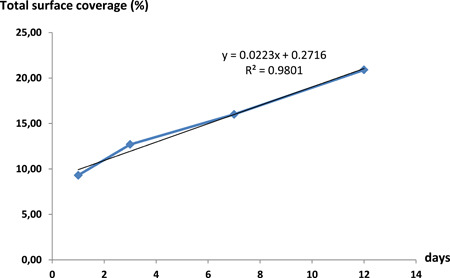
Evolution of the percentage of total surface coverage from Day 1 to Day 12 (methicillin resistant *Staphylococcus aureus* [MRSA] strain, duplicates).

The initial time‐course experiment highlights an increase in attached biomass over time which is illustrated by light microscopy pictures showing isolated microcolonies and bacteria on Day 1 and confluent microcolonies on Day 12 (Table 1). Moreover, the evolution of the percentage of total surface coverage over time follows a linear trend line with a regression coefficient of 0.98 and a slope of 2.23% (Figure 3). This seems close to the linear part of growth kinetics of oropharyngeal biofilms expressed as percentages of coverage as a function of time (Leonhard et al., 2018) but this will need to be investigated in future experiments.

**Table 1:** Time‐course experiment (MRSA strain, duplicates)

Time	Light microscopy picture^a^ (Crystal Violet staining)	MSC (cm^2^) ± SD (*N* = 20)	Percentage of total surface coverage^b^
Day 1	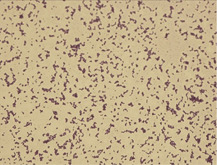	0.25 (±0.14)	9.3
Day 3	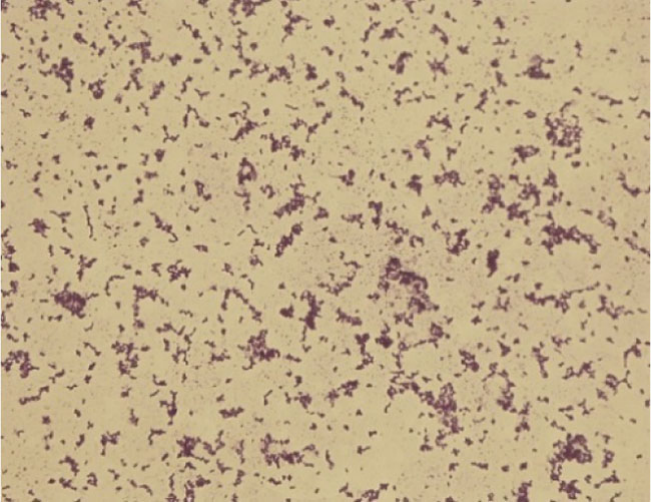	0.34 (±0.17)	12.7
Day 7	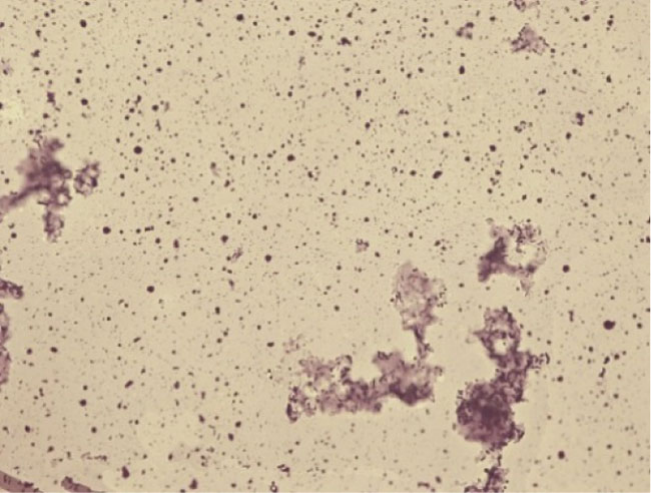	0.43 (±0.22)	16.0
Day 12	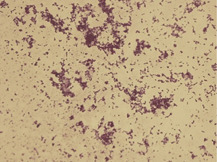	0.56 (±0.39)	20.9

Abbreviations: MSC, mean surface coverage; MRSA, methicillin resistant *Staphylococcus aureus*.

^a^ One representative picture and mean surface coverage are given for each day.

^b^ Total surface coverage = 2.68 cm² (see Table A3).

Due to the slow growth of the biofilm caused by our experimental conditions (nutrient‐poor medium, hydration and nutrient spray, and long drying phases), the experiments were stopped before a mature biofilm was obtained. The MSC measured on Day 12 was about 500 times higher than the detection limit (defined in Table A3) of the method and the biomass (adherent cells + extracellular polymeric substances) covered more than 20% of the total surface. In addition, we observed that Day 12 corresponded to the time needed to double the MSC measured on Day 1. For all these reasons, we considered the biomass obtained sufficient to perform further experiments.

**Figure 4:**
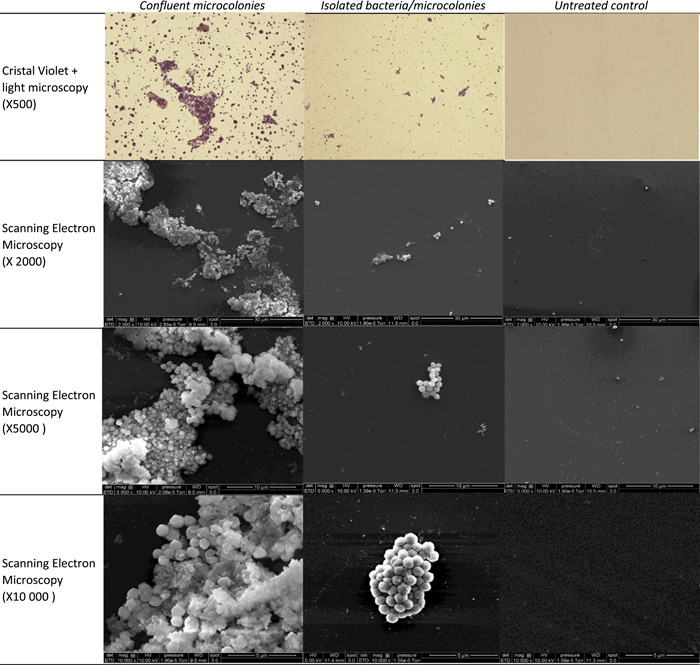
Dry‐surface biofilms obtained with methicillin resistant *Staphylococcus aureus* (MRSA) strain at the endpoint. One representative picture of a sample observed by light microscopy (×500) and scanning electron microscopy (×2000, ×5000, and ×10,000) is given. Focused is done on confluent microcolonies, and isolated microcolonies and bacteria.

Note, MRSA DSBs analyzed at the endpoint were made of isolated and confluent microcolonies embedded in an external matrix, which was confirmed by SEM observations (Figure 4). In this way, we obtained clusters of unevenly dispersed or confluent bacteria, as in hospital environmental DSBs (Ledwoch et al., 2019). No biomass was observed on untreated controls.

We chose to develop our method initially with a clinical strain of MRSA. Indeed, this species is very common in hospital environmental DSBs (Almatroudi et al., 2018; Hu et al., 2015; Vickery et al., 2012) and has been tested in all five biofilm models described in the literature (Almatroudi et al., 2015; Amaeze et al., 2020; Ledwoch et al., 2019; Nkemngong et al., 2020; Watson et al., 2018). The selected strain had previously shown an inherent ability to attach to inert surfaces under real‐life conditions, in this case, the inner surface of an indwelling catheter.

This method allowed the growth of MRSA DSBs. Under our experimental conditions, the total drying phase accounts for more than 91% of the total daily culture time, with a 2‐s hydration phase added to a 2‐h gradual desiccation phase under the laminar flow (considered as the total hydration phase) followed by a 22‐h dry‐holding phase. This is close to real‐life conditions.

We hypothesized that hydration phases were due to the propagation of respiratory secretions on the surfaces (Hu et al., 2015). Such secretions were mimicked by sterile enriched artificial saliva. We chose to spray the inoculum and SAS to simulate droplet dispersion of microorganisms and biological liquid which occurs during intubations, extubations, and aspirations. This technique allows for the random distribution of droplets on surfaces and suppresses turbulent flow and shear forces of the nutrient medium which exist in other models (Almatroudi et al., 2015; Amaeze et al., 2020; Nkemngong et al., 2020) but not in reality.

For biofilm analysis, MSC was chosen as the primary outcome as it gives an objective representation of surface conditions while allowing for biomass quantification (Table A3). Other authors assessed biofilms using surface coverage measurements (Flockton et al., 2019; Marion‐Ferey et al., 2003; Mountcastle et al., 2021) but the most widely studied criterion in the literature remains bacterial cultivability (Almatroudi et al., 2015; Amaeze et al., 2020; Ledwoch et al., 2019; Nkemngong et al., 2020; Watson et al., 2018). However, the absence of culturable bacteria in a biofilm does not mean that there is no biofilm because many phenotypic modifications occurring within the biofilm can transiently reduce cultivability (Fux et al., 2005). This parameter will be thoroughly studied in future experiments.

Finally, we determined the between‐run variation (reproducibility) and within‐run variation (repeatability) with MRSA strain to validate the method. As the ANOVA tests did not reach significance, neither between‐run nor within‐run variation was statistically demonstrated (Table 2). However, we note a relatively high coefficient of variation in both between and within‐run studies, which may be due to the manual spraying technique. Indeed, neither the spray itself nor the operator can deliver a perfectly repeatable volume. This represents the main limitation of this first version of our method. Our operating conditions allowed for MRSA strain to grow reproducible and repeatable DSBs that look like environmental DSBs.

**Table 2:** Between‐run and within‐run variations with MRSA strain; quantitative data and ANOVA *p* values

Strain	Between‐run variation*				Within‐run variation**			
	Mean MSC (cm²) (*N* = 6 runs)	SD	CV (%)	*p* (ANOVA)	Mean MSC (cm²) (*N* = 6 samples)	SD	CV (%)	*p* (ANOVA)
MRSA	0.57	0.21	36.8	0.10	0.65	0.24	36.9	0.10
Untreated Controls	<0.001	–		–	<0.001	–		–

Abbreviations: ANOVA, analysis of molecular variance; MSC, mean surface coverage; MRSA, methicillin resistant *Staphylococcus aureus*; SD, standard deviation.

* Between‐run variation: six different experiments with different operators, one single sample per experiment; individual MSCs measured from 10 random photos per sample; mean MSC and SD calculated from six individual MCSs.

** Within‐run variation: one single experiment with six different samples; individual MSCs measured from 10 random photos per sample; mean MSC and SD calculated from six individual MCSs.

### 3.2 Secondary objective: To apply the method to ESBL‐PE

Our method was applied to two ESBL‐PE, *Klebsiella pneumoniae*, and *Enterobacter cloacae*. Both had already shown their ability to attach to inert surfaces under real‐life conditions. The study of ESBL‐PE DSBs was of major interest to us because they are widespread in the hospital ecosystem of Martinique. Moreover, their persistence in DSBs has not yet been studied.

Both BLSE‐PE strains were able to grow DSBs using this spray method (Figure 5). They both consisted of isolated and confluent microcolonies embedded in an external matrix.

Figure 5Dry‐surface biofilms obtained with extended‐spectrum beta‐lactamase‐producing Enterobacteriaceae (ESBL‐PE) strains at the endpoint. (a) *Enterobacter cloacae*; (b) *Klebsiella pneumonia*. One representative picture of a sample observed by light microscopy (×500) and scanning electron microscopy (×2000, ×5000, and ×10000) is given. Focused is done on confluent microcolonies, and isolated microcolonies and bacteria.

**Figure:**
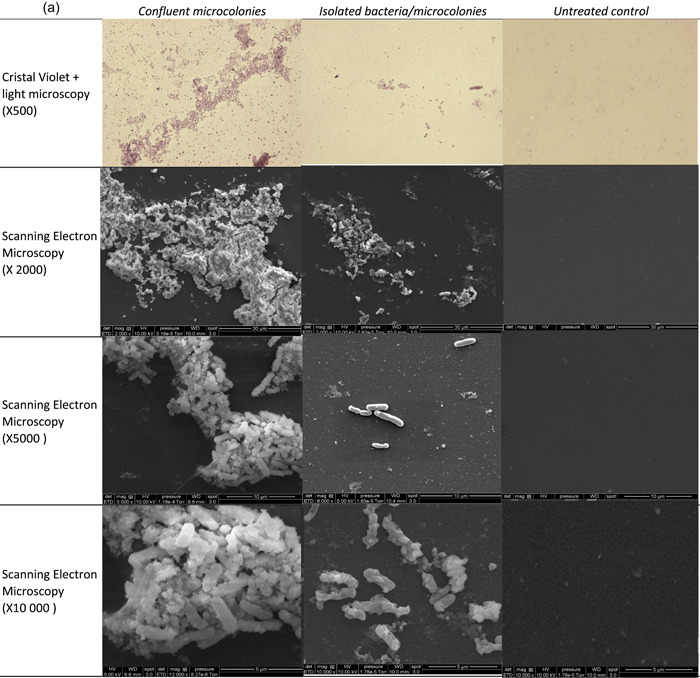

**Figure:**
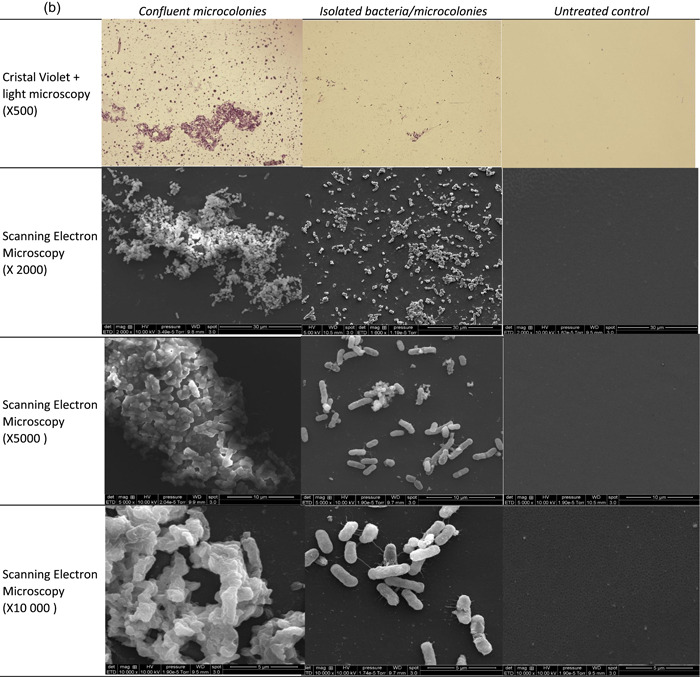

To confirm these conclusions based on microscopic observations, we studied interspecies variation by performing three independent experiments, one per strain, with six samples each. The MSCs (*N* = 6) obtained with each of the three strains were compared by ANOVA (Table 3). No significant difference in MSCs was observed (*p* = 0.11) indicating no interspecies variation.

**Table 3:** Interspecies variation

Strain	Interspecies variation		
	Mean MSC (cm²)	SD	CV (%)
	(*N* = 6 samples)		
MRSA	0.65	0.24	36.8
*Enterobacter cloacae*	0.47	0.11	23.4
*Klebsiella pneumoniae*	0.56	0.21	37.5
Untreated controls	<0.001	–	–
*p* (ANOVA)	0.11		

*Note*: Individual quantitative data and ANOVA *p*‐value.

Abbreviations: ANOVA, analysis of molecular variance; MRSA, methicillin resistant *Staphylococcus aureus*; MSC, mean surface coverage; SD, standard deviation.

Our operating conditions allowed for the growth of DSBs with *K. pneumoniae* ESBL and *E. cloacae* ESBL strains, which seemed to be similar to those obtained with MRSA in terms of appearance and surface coverage.

This pilot study using the manual spraying method we developed gave promising results and led us to launch a 3‐year research project called “Drysource” funded by the clinical research department of our hospital. This project includes the improvement of the spraying technique with the automation of the spraying using a nebulizer; the application of the method to numerous strains (in particular ESBL‐PE) including ATCC‐referenced strains of clinical and environmental origins with different resistance profiles and different attachment capacities (EPS producing/nonproducing strains); in‐depth study of bacterial cultivability and/or viability; and finally large‐scale trials on the effectiveness of cleaning products on the obtained DSBs.

## AUTHOR CONTRIBUTIONS

**Esther Christine**: Acquisition of data (lead); analysis and interpretation (equal); validation (equal); drafting the article (lead); final approval (equal). **Claude Olive**: Conception and design (supporting); validation (equal); revising the article critically (equal); final approval (equal). **Myriam Louisin**: Acquisition of data (supporting); validation (equal); revising the article critically (equal); final approval (equal). **Moustapha Drame**: Analysis and interpretation (equal); validation (equal); revising the article critically (equal); final approval (equal). **Karine Marion‐Sanchez**: Conception and design (lead); analysis and interpretation (equal); validation (equal); revising the article critically (equal); final approval (equal).

## CONFLICT OF INTEREST

The authors declare no conflict of interest.

## ETHICS STATEMENT

None required.

## Data Availability

The data generated and analyzed during this study are included in the published article.

## 1

**Table A1:** Composition of sterile artificial saliva (SAS) based on the ARTISIAL® drug formulation

Active substances	mg per 100 mL
Potassium chloride	62.45
Sodium chloride	86.55
Magnesium chloride	5.87
Calcium chloride	16.62
Dipotassium phosphate	80.32
Monopotassium phosphate	32.60

Excipients: Methyl parahydroxybenzoate, sodium carboxymethylcellulose, and sorbitol.

Magnesium chloride (Lavoisier, Laboratoires Chaix et du Marais) and calcium chloride solutions (Aguettant) were added to a phosphate‐buffered saline solution (Dubellco; Eurobio Scientific) to obtain the final concentrations in accordance with the formulation of Artisial®. The resulting solution was sterilized by filtration through a 0.22‐µm membrane. The excipients listed above were excluded and replaced by the addition of Brain Heart Infusion broth (20% v/v) to provide organic nutrients and viscosity.

**Table A2:** Growth of MRSA, ECLO, and KPN in BHI and enriched artificial saliva at H0 and after a 24‐h incubation time

Strain	Diluted inoculum (H0)		BHI 100% (H24)		Artificial saliva + BHI 20% (H24)	
	Turbidity	Viable cells^a^	Turbidity	Viable cells^a^	Turbidity	Viable cells^a^
		(Log_10_ CFU/mL)		(Log_10_ CFU/mL)		(Log_10_ CFU/mL)
MRSA	No	2.84 ± 0.03	Yes	8.26 ± 0.03	Yes	7.85 ± 0.02
ECLO	No	3.48 ± 0.03	Yes	9.11 ± 0.03	Yes	9.02 ± 0.05
KPN	No	3.45 ± 0.02	Yes	9.09 ± 0.02	Yes	8.91 ± 0.04

Abbreviations: BHI, Brain Heart Infusion; ECLO, *Enterobacter cloacae*; KPN, Klebsiella pneumoniae; MRSA, methicillin resistant *Staphylococcus aureus*.

^a^ Mean ± SD (*N* = 3).

**Table A3:** Coverage measurement using Scion Images softwareImage analysis using the “slice density” option and “analyze” tool

Step	Image	Description
*Step 1*: Original photograph (uncompressed TIFF format) after rinsing the surface and staining with crystal violet (light microscopy ×500)		Isolated sessile bacteria, isolated or confluent microcolonies appear in violet.
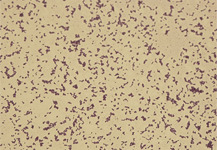
*Step 2*: The photograph converted into a red and gray image		The “density slice” option produces a black‐and‐white copy and uses a red/grayscale to separate the colored biomass from the surface background.
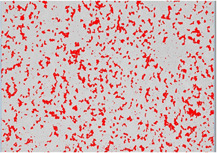
*Step 3:* Coverage measurement	0.27 cm^2^	The “analyze” tool measures exclusively the surface covered by the red‐colored biomass, expressed in cm². The final result is calculated by taking into account the magnification factor.

*Note*: Image analysis using the “slice density” option and “analyze” tool.

Each strain was inoculated in parallel in BHI broth and enriched artificial saliva and incubated at 37°C for 24 h. Culture media were examined for signs of turbidity and suspensions were enumerated at H0 and H24 by serial dilutions in sterile distilled water and spreading on Plate Count Agar. Experiments were performed in triplicates. The three strains grew both in BHI and enriched artificial saliva and reached a value ranging between 7.85 and 9.02 log_10_ CFU/mL.

Mean coverage is determined for one sample by measuring the coverage on 10 different photographs randomly chosen. The detection limit of this method is 0.001 cm² and represents the smallest red‐colored element allowing the measurement of a quantifiable area (different from zero) using the “analyze tool.” Conversely, the total surface coverage corresponds to 2.68 cm² (total red photograph). Detection limit = 0.05% of total surface coverage.
